# Occupational particle exposure and diabetes: a cohort study in Swedish construction workers

**DOI:** 10.1007/s00420-026-02224-4

**Published:** 2026-07-06

**Authors:** Karl Kilbo Edlund, Erik Sand, Martin Andersson, Sandra Johannesson, Björn Eliasson, Leo Stockfelt

**Affiliations:** 1 Occupational and Environmental Medicine, Sahlgrenska University Hospital, Region Västra Götaland, Gothenburg, Sweden; 2 Department of Occupational and Environmental Medicine, School of Public Health and Community Medicine, Institute of Medicine, Sahlgrenska Academy, University of Gothenburg, Box 414, 405 30 Gothenburg, Sweden; 3 Department of Epidemiology and Global Health, Umeå University, Umeå, Sweden; 4 Department of Medical Sciences, Sahlgrenska Academy, University of Gothenburg, Gothenburg, Sweden

**Keywords:** Diabetes mellitus, type 2, Occupational exposure, Air pollutants, occupational, Particulate matter, Construction industry

## Abstract

**Purpose:**

Diabetes is a growing global health concern. Environmental exposure to airborne particles has recently been identified as a risk factor for type 2 diabetes. Despite hundred- or thousandfold concentrations in many occupational settings, epidemiological studies in workers are very scarce. This study aims to quantify the association between occupational inorganic particle exposure and incident type 2 diabetes among Swedish construction workers.

**Methods:**

This was a retrospective cohort analysis in the Swedish Construction Workers’ Cohort (Bygghälsokohorten), with data from occupational health examinations between 1971 and 1993. Exposure assessment was based on a cohort-specific job-exposure matrix. Data from the National Patient Register, the National Diabetes Register, and the National Prescribed Drug Register were combined to identify incident type 2 diabetes cases. Longitudinal, multivariable Cox proportional hazards regression models were fit to estimate adjusted hazard ratios (aHR).

**Results:**

We included 275,941 male workers with a mean follow-up time of 31 years. Slightly more than half were exposed to inorganic dusts or fumes. In crude analyses, workers exposed to inorganic dust and fumes were at an increased risk of type 2 diabetes. After confounder adjustment, only workers exposed to high levels of cement, concrete, and quartz dust were associated with an increased risk of type 2 diabetes (strongest for high levels of cement dust, aHR 1.12, 95% CI 1.02‒1.24).

**Conclusions:**

In this large cohort of construction workers with long follow-up, workers with high exposure to cement, concrete, and quartz dust were at a moderately increased risk of type 2 diabetes.

**Supplementary Information:**

The online version contains supplementary material available at 10.1007/s00420-026-02224-4.

## Introduction

Occupational particle exposure remains common (Grahn et al. 2021; Sadhra et al. 2020) and has been linked to multiple adverse health effects, including increased risk of malignant and non-malignant pulmonary disease (Bergdahl et al. 2004; Grahn et al. 2021; Torén and Järvholm 2014), and cardiovascular diseases (Fang et al. 2010; Gustavsson et al. 2001; Mocevic et al. 2015; Torén et al. 2007). The Global Burden of Disease collaboration has estimated that occupational particle exposure causes at least 590,000 premature deaths every year (Brauer et al. 2024). However, evidence for impacts on metabolic diseases is scarce.

Growing evidence suggests that ambient particle exposure is a risk factor for type 2 diabetes (Eze et al. 2015; Yang et al. 2020). A cross-sectional study combining ambient and occupational exposure in the population-based UK Biobank, on the other hand, found no associations (Dimakakou et al. 2020). Controlled human exposure studies and animal experiments have shown that inhaled particles initiate oxidative stress and low-grade inflammation (Andersen et al. 2021; Gangwar et al. 2020). Oxidative stress and inflammation drive type 2 diabetes through insulin resistance and beta cell dysfunction (Kahn et al. 2014; Tsalamandris et al. 2019). Esposito and colleagues (2016) suggested three main biological pathways for ambient particles: endothelial dysfunction reducing cellular glucose uptake, dysregulation of visceral adipose tissue, and increased hepatic insulin resistance. Epidemiological studies are necessary to determine the relevance of these mechanisms in real-life workplace settings.

Studies of occupational particle exposure and diabetes remain scarce, despite considerably higher particle exposures (Viitanen et al. 2017) and a temporally intermittent exposure pattern determined by task and working hours rather than meteorology. Increases in inflammatory markers have been shown after particle exposure in various occupational settings (Kim et al. 2005; Ohlson et al. 2010; Westberg et al. 2016). A cohort study in South Korea (Yun et al. 2022) suggested a strong relationship between mineral dust exposure and diabetes risk; however, this study was limited by a relatively short follow-up. The objective of this study was to investigate the association between occupational particle exposure and incident type 2 diabetes. We hypothesised that exposure to inorganic dusts, particularly mineral dusts, would be associated with an increased risk of type 2 diabetes.

## Methods

We performed a retrospective cohort study. Ethical permission for this study was granted by the Swedish Ethical Review Authority (Dnr 2024-02083-02).

### Study population

Established in 1968, *Bygghälsan* was a comprehensive occupational health service jointly organised by employers and labour unions, and financially supported by the government. Worker participation was voluntary, reaching an approximate 80% participation rate (Stattin and Järvholm 2005). From 1971 and until the discontinuation of *Bygghälsan* in 1993, occupational codes as well as basic health data from consenting workers were entered into a registry for use in research. The Swedish Construction Workers’ Cohort (*n* = 389,132) has been extensively described elsewhere (Bergdahl et al. 2004; Kilbo Edlund et al. 2024).

Information on the participants’ baseline status (height, weight, and smoking habits) was obtained from the first recorded health examination. In case this information had not been recorded, the first available information from a subsequent examination was used. Body mass index (BMI) was calculated from height and weight measures taken at the baseline examination. Questionnaire data on cigarette, cigar, and pipe smoking were obtained until 1974 and after 1978 (questions on smoking were not included in questionnaires 1975‒June 1978). For current and former smokers, we *a priori* classified the combined cigarette, cigar, and pipe smoking intensity as light (equivalent to 0‒14 cigarettes per day), medium (15‒24 cigarettes per day) or heavy (≥ 25 cigarettes per day).

Migration, employment, residence, and civil status data were obtained from Statistics Sweden. Socioeconomic classification (SEI) was derived from the National Swedish Census Data of 1980 and 1985, based on the skill level of the individual’s main employment. Workers were classified as non-skilled manual workers, skilled manual workers, non-manual workers, self-employed workers, or farmers. Missing instances in the 1985 data were supplemented with data from 1980 if available. Workplace codes for all participants were obtained from the longitudinal integrated database for health insurance and labour market studies (LISA) and total population register (RTB), available from 1985. For each year, each participant was classified as working (with a workplace code) or non-working (without a workplace code, mostly retired).

### Exposure assessment

A job-exposure matrix (JEM) was constructed by expert occupational hygienists who performed a series of site visits during 1971‒77 (Bygghälsan, 1977; Lee et al. 2003). Originally, the JEM included 214 job codes; from 1985, titles with similar exposures were grouped, resulting in 90 job codes. Occupational exposures were classified for each job code as either unexposed or exposed, with the latter graded on a scale from 1 to 5. For diesel exhaust, a 0.5 grade was retrospectively added (Lee et al. 2003). Grades ≥ 3 represented exposure exceeding the occupational exposure limit (OEL) at the time or, in the absence of an OEL, levels above what was considered acceptable at the time. An overview of relevant OEL in Sweden at the time of the construction of the JEM is available in a previous publication (Kilbo Edlund et al. 2024).

We identified nine particle exposures (cement dust, concrete dust, quartz dust, welding fumes, diesel exhaust, asphalt fumes, wood dust, asbestos, and manmade mineral fibres [MMMF]), which were grouped into three binary exposure categories: *inorganic dusts or fumes* (cement dust, concrete dust, quartz dust, welding fumes, diesel exhaust, or asphalt fumes), *wood dust*, and *fibres* (asbestos or MMMF). Some participants were included in more than one of the three exposure categories. Lastly, for exposure-response analyses, exposure to each of the nine particle types was graded as low (grade 1, or 0.5 for diesel exhaust) or high (grade ≥ 2, or ≥ 1 for diesel exhaust).

### Outcome data collection

Incident diabetes was identified from national register data using the Swedish version of the International Classification of Disease (ICD) codes (ICD-8 and ICD-9 code 250, ICD-10 codes E10‒E14) from the National Patient Register (NPR) for inpatient (1987‒) and outpatient (2001‒) care. We did not classify diabetes type based on the ICD codes since different codes were introduced only with ICD-10 and remained inconsistent in the early years of implementation. Instead, to improve case identification and incidence timing, we obtained additional data from the National Diabetes Register (NDR, 1996‒) and the National Prescribed Drug Register (LMED, 2005‒). From the NDR we obtained information on diabetes type, treatment, and year of onset. The NDR (Gudbjörnsdottir et al. 2003) contains diabetes cases reported from both primary and secondary care. Date of first prescription of any antidiabetic medication (Anatomical Therapeutic Chemical [ATC] code A10) was extracted from the LMED. Incident diabetes was defined as first registered diabetes diagnosis, year of onset registered in the NDR, or the first prescription of antidiabetic medication.

### Data analysis

All workers were included for whom occupational title had been registered at the baseline examination and for whom complete data could be obtained (89% of the original cohort, see study flowchart in Figure S1). We excluded female workers (5%), as few were employed in the exposed occupations. We excluded workers younger than 15 or older than 65 years at first examination (1%). We also excluded individuals for whom no smoking or anthropometry data were available (8%), office workers (5%), and individuals with reused personal identification numbers (< 0.1%). Lastly, we excluded 2692 workers with type 1 diabetes (0.7%). We thus included 288,529 workers (74% of the original cohort). Among these, 12,588 (3% of the original cohort) did not contribute any risk time (due to death or emigration before 1987 or before age 20 years), leaving data from 275,941 workers retained for further analysis.

To quantify the association between occupational particle exposure and incident diabetes we fit multivariable Cox proportional hazards models with calendar year as the time axis using a counting process formulation to account for delayed entry, with follow-up commencing at the individual-specific entry time, i.e., the first calendar year after baseline examination, the year the worker turned 20 years old, or 1987, the first year with complete healthcare records, whichever occurred last. Workers were censored at emigration, the year they turned 90 years old, or death. The regression formula used was $\:h\left(t\mathbb{\:}|\mathbb{\:}\mathbb{Z}\left(t\right)\right)={h}_{0}\left(t\right)\cdot\:\mathrm{exp}({\beta\:}_{E}\mathbb{E}+{\beta\:}_{Z}\mathbb{Z}\left(t\right))$, where $\:h\left(t\mathbb{\:}|\mathbb{\:}\mathbb{Z}\right)$ signifies the hazard function at time $\:t$ conditional on the set of covariates $\:\mathbb{Z}\left(t\right)$, $\:{h}_{0}\left(t\right)$ the baseline hazard over time, $\:\mathbb{E}$ the set of exposure variables included in the analysis, and $\:{\beta\:}_{\mathbb{E}}$ and $\:{\beta\:}_{\mathbb{Z}}$ the respective sets of slope coefficients. In the primary analyses, the set $\:\mathbb{E}$ included the three exposure categories: inorganic dusts and fumes, wood dust, and fibres. In secondary analyses, $\:\mathbb{E}$ instead included the separate nine particle types. All models were specified as multipollutant models, as pairwise Spearman correlations between particle types were below 0.8 and the variance inflation factor (VIF) was < 5 for all exposures. In the exposure-response analyses, $\:\mathbb{E}$ included all separate nine particle types, each graded as none, low, or high exposed. Exposure-response trends were tested by modelling these categories as continuous variables. Three different sets of covariates, $\:\mathbb{Z}\left(t\right)$, were used. In a crude model, we included only age (natural spline with four degrees of freedom). In the main model, we added smoking status and intensity at baseline (both categorical), and baseline BMI (natural spline with four degrees of freedom). In the extended model, we also added civil status (yearly updated from register data) and socioeconomic status.

The proportional hazards assumption was probed by visual inspection of the Schoenfeld residuals. Sensitivity analyses were performed to ensure the robustness of the modelling strategy. Firstly, workers who were classified as exposed to more than one of the three exposure categories were excluded. Secondly, analyses were stratified by region. However, since only 1.5% of the cohort was born outside the Nordic countries, analyses were not stratified on immigration status. Finally, the main analyses were repeated with age as the time axis and calendar year modelled using a natural spline (four degrees of freedom). Formal correction for multiple comparisons was not implemented, as the analyses were guided by *a priori* hypotheses and such approaches may increase the risk of type II error. All calculations were performed in R v 4.1.1 using the *survival* package (R Core Team 2021; Therneau and Grambsch 2000).

## Results

Baseline characteristics of the workers are presented in Table 1. Most workers had their first recorded health examination during the 1970s. The mean age at baseline was 33 years and differed little between exposure categories. Around 40% were active smokers at baseline, of whom 6% were categorised as heavy smokers. Smoking was slightly more common among workers exposed to inorganic dusts and fumes and among workers exposed to fibres. The mean BMI was around 24 in each exposure category, but overweight and obesity were more common among workers with inorganic dusts or fumes exposure. Similarly, there were socioeconomic differences, with 12% of unexposed workers classified as unskilled manual workers compared to 20% among those exposed to inorganic dusts or fumes or exposed to wood dust (Table S1).

**Table 1:** Baseline characteristics of the study population

*n*	All	Unexposed	Inorganic dusts or fumes	Wood dust	Fibres
	275,941	103,225	150,847	18,894	22,005
Age, mean (SD)	33.3 (12.6)	32.1 (11.7)	34.2 (13.0)	32.4 (13.0)	31.8 (12.5)
Current smokers, %	40	37	43	36	44
Heavy smokers*, %	6	6	7	5	6
Year of first visit, mean (SD)	1979 (7)	1980 (7)	1979 (7)	1980 (7)	1979 (6)
BMI, mean (SD)	24.1 (3.1)	23.9 (3.0)	24.2 (3.2)	23.8 (2.9)	23.8 (3.0)
Overweight, %	29.6	27.9	31.2	27.3	27.1
Obese, %	4.2	3.6	4.8	2.9	3.6
Unskilled manual workers**, %	17	12	20	20	16
Unmarried at age 50***, %	24	22	25	24	24

n, number; SD, standard deviation; BMI, body-mass index *Combined cigarette, cigar and pipe smoking equivalent to ≥ 25 cigarettes per day **Based on socioeconomic classification (SEI) 1985 ***Civil status analysed as a time-varying variable, shown here at age 50 for descriptive purposes

A detailed breakdown of exposure is presented in Table 2. Just over a third (37%) of the workers were categorised as unexposed, half (55%) to inorganic dusts or fumes, 7% to wood dust, and 8% to fibres. Only 19 030 workers (11% of the exposed) were included in more than one of the three main exposure categories. The most common particulate exposure was concrete dust, followed by quartz dust. Just over a third (35%) were classified as high exposed to any of the nine particle types. Exposure to asphalt fumes was rare and all exposed workers were classified as high exposed. Correlations between exposure to different particle types were generally low to moderate (Figure S2).

**Table 2:** Number of cohort participants distributed across nine particle types

Exposure	Unexposed	Low exposed	High exposed
*Inorganic dusts or fumes*			
Cement dust	253,060	19,332	3549
Concrete dust	169,876	52,389	53,676
Quartz dust	219,760	46,429	9752
Welding fumes	248,745	1163	26,033
Diesel exhaust	238,826	23,427	13,688
Asphalt fumes	270,527	–	5414
*Wood dust*			
Wood dust	257,047	18,240	654
*Fibres*			
Asbestos	263,491	8169	4281
MMMF	254,128	13,649	8164

MMMF, man-made mineral fibres

Over a median follow-up time of 31 years, we detected 40,583 incident cases of type 2 diabetes. Incident cases per calendar year, age year, and registry source are shown in Figure S3. Person-time by age, employment status, and exposure group are shown in Figure S4. The total number of person-years was 7,463,733, resulting in a crude incidence rate of 5.4 cases per 1000 person-years. The median age at diagnosis was 63 years among the unexposed, inorganic dusts or fumes exposed, and wood dust exposed, while it was 62 years among the fibre exposed. While most cases were identified from the NDR, the LMED became the predominant source of outcome identification over the last decades of follow-up. Cases identified from the NPR tended to be somewhat older at diagnosis than cases identified from the two other registers.

We did not see any increased risk of type 2 diabetes among workers exposed to inorganic dusts or fumes after adjustment for baseline risk factors (i.e., age, smoking, and BMI; see Table 3). While the hazard was increased among workers exposed to inorganic dusts and fumes in the crude model (accounting only for age), this was explained by the higher baseline BMI among the exposed. After further adjustment for socioeconomic category and civil status, in the extended covariate model, there was a borderline association among workers exposed to fibres. After removal of BMI from the extended model, exposure to inorganic dusts was again associated with diabetes (see Table S2). Excluding workers who were classified into more than one of the three exposure categories or adding further adjustment for region of examination had no effect on the estimates (Table S2). Reanalysis with age as the time axis yielded numerically identical estimates. Visual inspection of the Schoenfeld residuals indicated no violations of the proportional hazards assumption for the main exposures, whereas the effect of baseline BMI on disease risk attenuated over time, consistent with expectations.

**Table 3:** Associations between occupational particle exposure (three main groups) and incident type 2 diabetes mellitus

Exposure		Cases			
	Model	Unexposed	Exposed	Person-years	aHR (95% CI)
Inorganic dusts or fumes	Crude	15,165	22,689	7,463,733	1.08 (1.05–1.10)
	Main	15,163	22,682	7,462,230	1.01 (0.99–1.03)
	Extended	13,949	20,507	6,172,017	1.02 (0.99–1.04)
Wood dust	Crude	15,165	2311	7,463,733	0.92 (0.88–0.96)
	Main	15,163	2310	7,462,230	0.95 (0.91–0.99)
	Extended	13,949	2032	6,172,017	0.95 (0.91–1.00)
Fibres	Crude	15,165	3221	7,463,733	0.98 (0.95–1.02)
	Main	15,163	3220	7,462,230	1.02 (0.99–1.06)
	Extended	13,949	2963	6,172,017	1.04 (1.00–1.08)

aHR, adjusted hazard ratio; CI, confidence intervalThe crude model included only age. The main model added smoking status and intensity at baseline, and baseline BMI. The extended model added civil status and socioeconomic status.

In the exposure-response analyses, high exposure to cement, concrete, and quartz dust was associated with an increased risk of diabetes, with statistically significant trends across exposure categories detected for concrete and quartz dust (see Table 4, see number of cases and person-year in Table S3). Only low exposure to welding fumes and asbestos appeared to be associated with an increased risk of diabetes, with no statistically significant risk elevations among high-exposed workers.

**Table 4:** Associations (aHR, 95% CI) between nine particle types and incident type 2 diabetes, estimated from multipollutant models adjusted for the covariates in the main covariate model, for binary exposure and where particle exposure was graded as low or high, and *p*-value for trend between no, low, or high exposure

Exposure	Any exposure	Low exposure	High exposure	*p*_trend_
Cement dust	0.97 (0.93–1.01)	0.96 (0.91-1.00)	1.12 (1.02–1.24)	0.232
Concrete dust	1.02 (0.99–1.04)	0.96 (0.93–0.99)	1.06 (1.03–1.09)	0.007
Quartz dust	1.02 (0.99–1.05)	1.02 (0.98–1.06)	1.06 (1.00-1.11)	0.022
Welding fumes	0.97 (0.93–1.01)	1.11 (0.95–1.29)	1.00 (0.96–1.05)	0.075
Diesel exhaust	1.01 (0.98–1.04)	1.01 (0.98–1.05)	0.94 (0.89–0.99)	0.947
Asphalt fumes	0.94 (0.88–1.01)	–	0.97 (0.91–1.04)	0.114
Wood dust	0.95 (0.91–0.99)	0.94 (0.90–0.99)	1.10 (0.90–1.35)	0.062
Asbestos	1.11 (1.03–1.19)	1.20 (1.11–1.30)	1.06 (0.93–1.21)	0.034
MMMF	0.96 (0.91–1.01)	0.96 (0.90–1.02)	0.90 (0.81-1.00)	0.139

aHR, adjusted hazard ratio; CI, confidence interval; MMMF, man-made mineral fibresThe crude model included only age. The main model added smoking status and intensity at baseline, and baseline BMI. The extended model added civil status and socioeconomic status

## Discussion

In this large cohort we found a 12% increased risk of type 2 diabetes among construction workers with high exposure to cement dust, with 6% increases among workers with high exposure to concrete or quartz dust. Risk increases after exposure to other inorganic particles were null when accounting for the higher BMI among the exposed workers. No consistent risk elevations were found among workers with wood dust or fibre exposure, where there were also considerably fewer workers. These results lend partial support for our hypothesis. Given that particle exposure is common in many occupational settings, also the rather moderate increase in the risk of diabetes could have a public health impact.

The association with high exposure to cement, concrete, and quartz dust aligns with the cohort study among South Korean factory workers (Yun et al. 2022), reporting an elevated risk of diabetes among workers exposed to mineral dust (aHR 1.66, 95% CI 1.26–2.20). Since JEMs were used for exposure assessment, no concentration data are available in either study to compare the exposure levels. Nonetheless, only 16% of the South Korean cohort was classified as exposed, compared to 55% in our study. This difference could indicate higher particle exposure among construction workers than factory workers, or that their exposure group may be a more selected, highly exposed group, aligning with their higher aHR.

In the age-adjusted crude model, exposure to inorganic dusts and fumes was associated with an increased risk of diabetes. This association was attenuated after adjustment for BMI, indicating that BMI is an important confounding factor. Construction workers have a higher prevalence of overweight and obesity than the general population, likely reflecting a combination of occupational, lifestyle, and socioeconomic influences (Strickland et al. 2017). Workers were not unexposed at baseline, and emerging evidence from ambient settings suggests that particle exposure may contribute to the development of obesity (Munir et al. 2025). This raises the possibility that BMI may partly lie on the causal pathway, giving it an ambiguous role as both a confounder and, likely to a lesser extent, a mediator. Adjustment for occupational skill level, used as a proxy for socioeconomic status, did not materially change the estimates and did not account for the confounding effect of BMI. The attenuation after BMI adjustment is consistent with null findings from a cross-sectional study in the UK Biobank (Dimakakou et al. 2020).

Different pathomechanisms have been suggested to explain the association between particle exposure and diabetes, however specific evidence for occupational particle exposures remains scarce. In mice, prolonged exposure to ambient particulate air pollution can exacerbate vascular and adipose inflammation, visceral adiposity, and insulin resistance, which are hallmarks of type 2 diabetes mellitus (Sun et al. 2009). Rao and colleagues suggested four pathomechanisms for the diabetic effects of particle exposure: (i) an inflammatory response to particle exposure exacerbating insulin resistance; (ii) endoplasmic reticulum stress, in which un- or misfolded proteins accumulate cellular dysfunction; (iii) effects on glucose and lipid metabolism, where particle exposure may upset the balance between white and brown adipose tissue; and (iv) activation of the central nervous system, which may contribute to metabolic dysregulation (Rao et al. 2015). Prolonged particle exposure may also induce steatosis, which affects the hepatic glycogen balance and may precede diabetes onset (Tsalamandris et al. 2019; Zheng et al. 2013). Lastly, type 2 diabetes is part of the metabolic syndrome, with tentative evidence for a link to ambient particle exposure (Dai et al. 2024) but with very limited evidence in the occupational setting. To our knowledge, only one study has examined metabolic biomarkers in relation to occupational dust exposure, reporting cross-sectional associations between respirable silica exposure and elevated levels of adiponectin, adipsin, and resistin (Sauni et al. 2012).

There are several important strengths of this study, including the cohort’s size and long follow-up time as well as the high quality and completeness of the registry data. Two major advantages of the NDR are that it includes the retrospectively reported year of diabetes onset, which can predate the date of registration, and that it provides a reliable classification of diabetes type. The registry-based follow-up, independent of occupational status, reduces the risk of a healthy worker effect. Important limitations include the JEM-based exposure assessment, with risk of exposure misclassification, and lack of information on exposure duration and task changes. The JEM-based approach was necessary due to the cohort’s age and size and the paucity of workplace measurements but may risk attenuating any true effects. Nonetheless, Swedish construction workers are known to be stable in their occupation (Bergdahl et al. 2004; Kilbo Edlund et al. 2024); this was also corroborated in our analyses using Statistics Sweden’s workplace coding. Socioeconomic and lifestyle data in this cohort are limited, precluding fuller control for potential confounding, including differential diagnostic delay and lifestyle-related factors such as physical activity, diet, and alcohol consumption. However, the influence of these lifestyle factors on exposure is likely largely mediated by socioeconomic status. Moreover, the cohort was relatively socioeconomically homogeneous, particularly after excluding office workers. In extended models, additional adjustment for skill level and civil status had negligible impact on the estimates.

The slightly inverse associations for wood dust might nonetheless signal residual confounding, and similar patterns have been seen in previous studies of this cohort (Blanc et al. 2015; Kilbo Edlund et al. 2024). While exposure was defined at baseline and follow-up continued regardless of working status, some degree of healthy worker survivor bias cannot be excluded, as prior health status may have influenced occupational selection and affected exposure status at baseline. Furthermore, competing risk from death may have underestimated the diabetes incidence among the exposed, potentially biasing the observed associations towards the null. Although mortality was modestly elevated in the exposed group, the impact of this bias on the estimates is likely limited. The marginal or inconsistent findings for some specific dust types may reflect multiple testing, residual confounding or selection effects, and should be interpreted with caution. Finally, reliance on register-based diabetes ascertainment, particularly during the early study period, may have resulted in delayed case identification until contact with hospital-based care occurred. This limitation was addressed in part by incorporating data from the NDR (from 1996), which includes retrospectively assigned year of diagnosis and achieves high population coverage, and in the later period by use of data from the LMED.

Further studies are needed to corroborate these findings; to evaluate the causality, particularly in relation to obesity and other components of the metabolic syndrome; and to design mitigation strategies. Compelling evidence could be obtained if it were possible to detect early pre-diabetic conditions and, possibly, to reverse these with exposure reductions through workplace improvements. If workplace particle levels are measured, which has not been the case in either of the previous studies of occupational particle exposure and diabetes, these findings could evaluate the need of revising current OEL. While notable improvements in the working environment have been achieved since the 1970’s, exposure reductions may have lost pace over the recent decades, with workers in small and medium-sized enterprises, in the informal sector, or under precarious conditions being particularly vulnerable (Gustavsson et al. 2022). Renewed efforts aimed at improving the work environment are therefore motivated.

## Conclusion

In this large cohort study of Swedish construction workers, long-term exposure to high levels of cement, concrete, and quartz dust was associated with a modestly increased risk of type 2 diabetes.

## Supplementary Information

Below is the link to the electronic supplementary material.

Supplementary Material 1.

## Data Availability

All statistical code developed for this study is publicly available via GitHub (https://github.com/kilboedlund/podm). Cohort and register data are available from Umeå University and Swedish registry holding authorities, respectively, for researchers with appropriate ethical permissions.
