# Supplementary material for: Occupational particle exposure and diabetes: a cohort study in Swedish construction workers
Source: Int Arch Occup Environ Health. 2026 Jul 6;99(5):30. doi: 10.1007/s00420-026-02224-4 (PMC13337675; doi:10.1007/s00420-026-02224-4)
Supplement: Supplementary file 1 — Supplementary Material 1. [file 420_2026_2224_MOESM1_ESM.docx]

SUPPLEMENTARY FIGURES AND TABLES

# Occupational particle exposure and diabetes: A cohort study in Swedish construction workers

Karl Kilbo Edlund^1,2^, Erik Sand^2^, Martin Andersson^3^, Sandra Johannesson^1,2^, Björn Eliasson^4^, Leo Stockfelt^1,2^

*
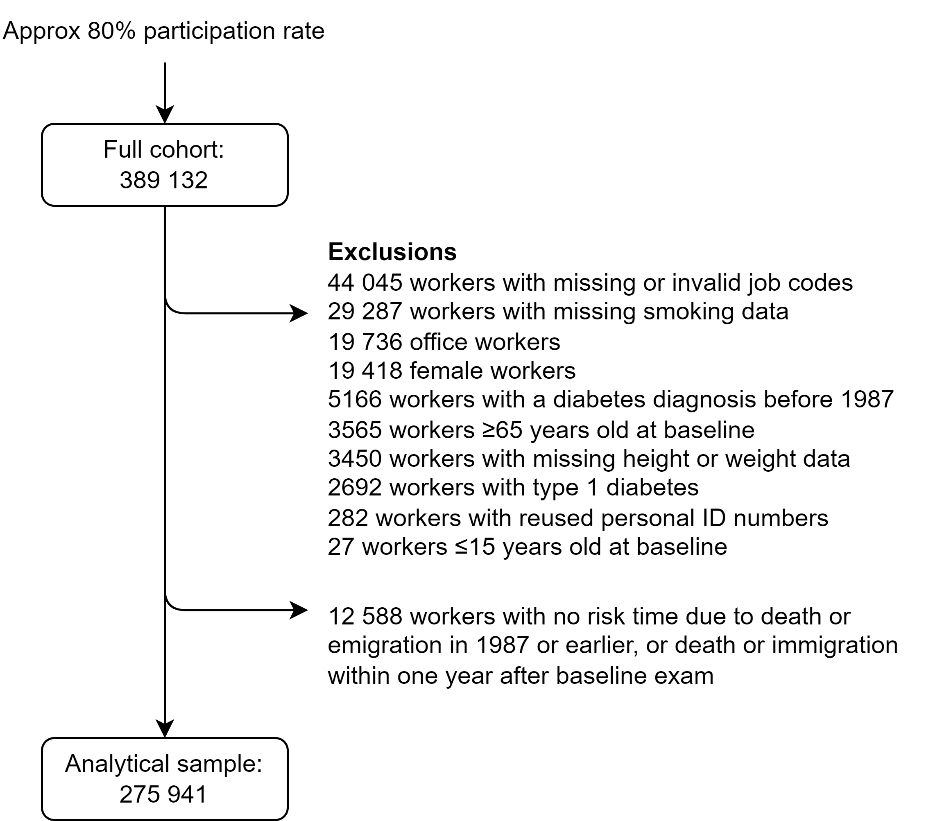
*

*Figure S1. Study flowchart.*

*
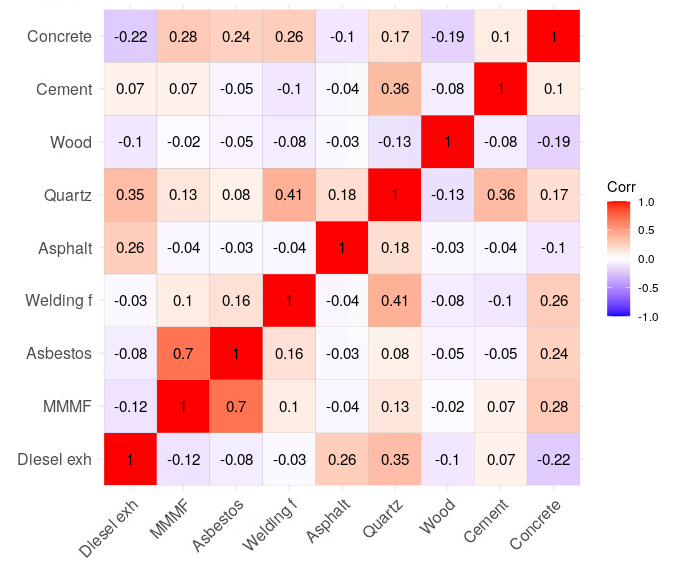
*

*Figure S2. Spearman correlation coefficients for between exposure to different particle types (graded as none, low, or high).*


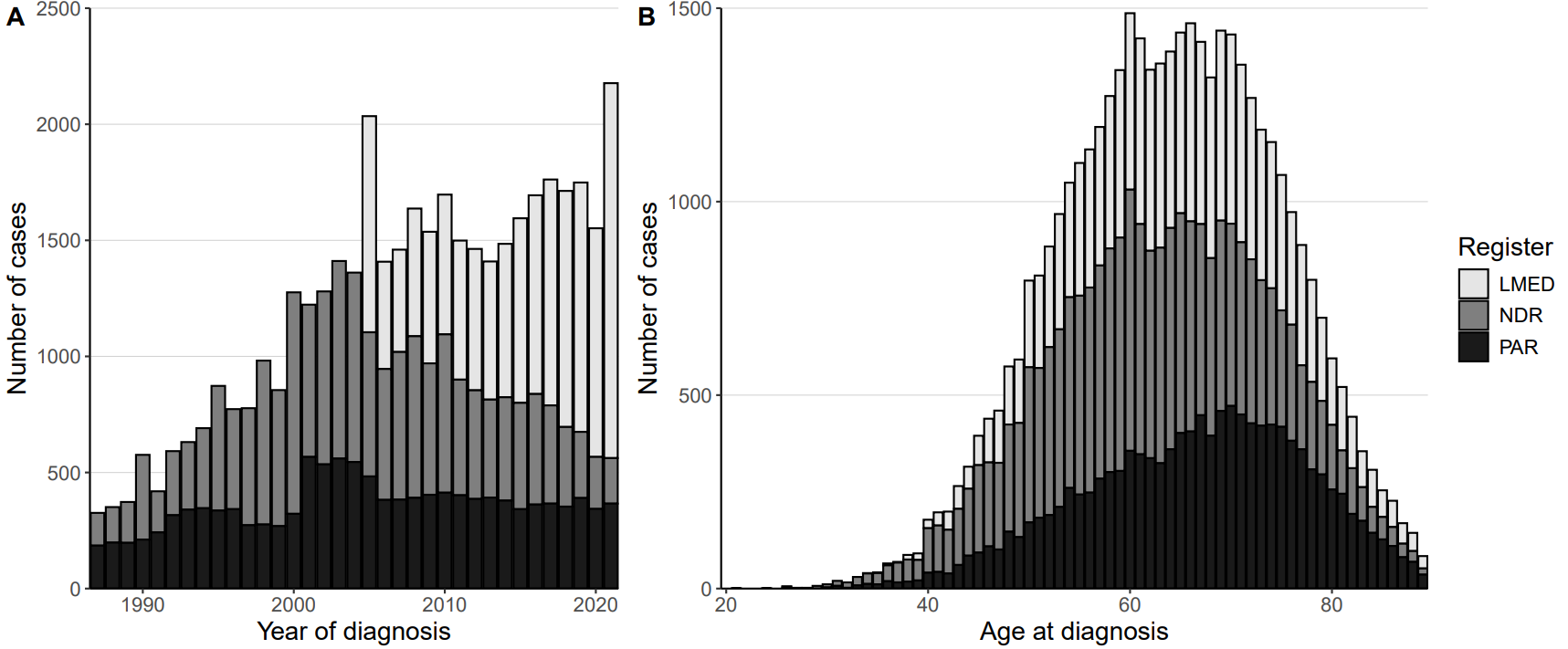
*Figure S3. Incident cases of type 2 diabetes in the study population during the follow-up period, categorised by the register in which they were first identified (LMED, National Prescribed Drug register; NDR, National Diabetes Register; PAR, National Patient Register).* ***A)*** *Incident cases by year of diagnosis.* ***B)*** *Incident cases by age at diagnosis.*


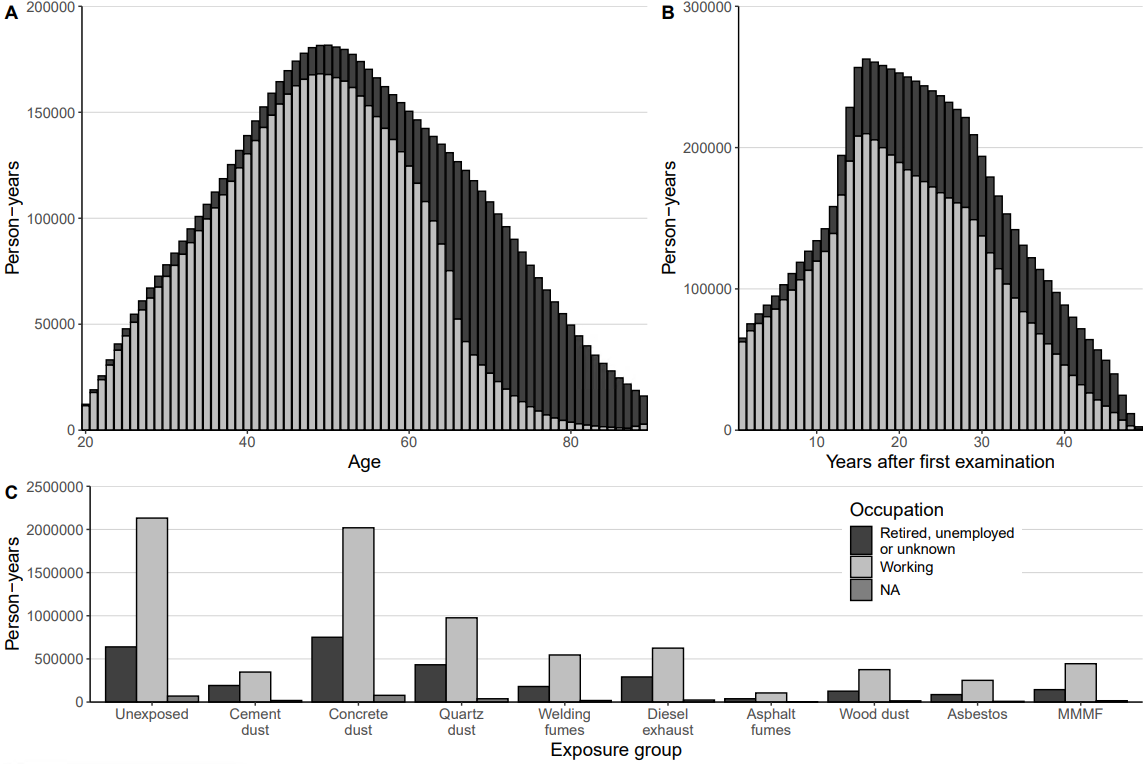
 *Figure S4. Occupational group of participants in the cohort, obtained from Statistics Sweden’s employment register (note that workers not yet recruited into the cohort as well as deceased individuals are not included in the diagrams).* ***A)*** *Occupational group by age.* ***B)*** *Occupational group by years since cohort inclusion.* ***C)*** *Particle exposure by occupational group, allowing multiple exposures.*

*Table S1. Breakdown of SEI categories across exposure groups (three main groups).*

| **SEI category** | **All** | | **Unexposed** | | **Dusts** | | **Wood dust** | | **Fibres** | |
| --- | --- | --- | --- | --- | --- | --- | --- | --- | --- | --- |
|  | **N** | **%** | **N** | **%** | **N** | **%** | **N** | **%** | **N** | **%** |
| **Manual worker, unskilled** | 37305 | 17% | 10329 | 12% | 23622 | 20% | 2803 | 20% | 2932 | 16% |
| **Manual worker, skilled** | 139888 | 63% | 46600 | 55% | 82261 | 68% | 9292 | 66% | 13280 | 72% |
| **Farmer** | 1497 | 1% | 430 | 1% | 931 | 1% | 114 | 1% | 101 | 1% |
| **Non-manual worker** | 31920 | 14% | 22296 | 26% | 8387 | 7% | 1021 | 7% | 1372 | 7% |
| **Self-employed** | 10779 | 5% | 4561 | 5% | 5289 | 4% | 786 | 6% | 805 | 4% |
| **Self-employed academic** | 17 | 0% | 14 | 0% | 2 | 0% | 0 | 0% | 1 | 0% |
| **Missing** | 54536 |  | 18996 |  | 30355 |  | 4878 |  | 3514 |  |

*Table S2. Associations* (*aHR (95% CI)) between occupational particle exposure (three main groups) and incident type 2 diabetes mellitus, (a) adjusted for all covariates in the extended model except BMI, (b) excluding participants with multiple exposures, and (c) with additional adjustment for geographical region of examination.*

| Exposure | Not adj. for BMI | Excl. multiple | Adj. for region |
| --- | --- | --- | --- |
| Inorganic dusts or fumes | 1.06 (1.03, 1.08) | 1.01 (0.99, 1.03) | 1.01 (0.99, 1.03) |
| Wood dust | 0.91 (0.87, 0.95) | 0.94 (0.90, 0.98) | 0.95 (0.91, 0.99) |
| Fibres | 0.99 (0.95, 1.03) | 0.93 (0.85, 1.03) | 1.02 (0.98, 1.06) |
| aHR, adjusted hazard ratio; CI, confidence interval | | | |

*Table S3. Number of cases and person-years for the exposure-response analyses.*

| **Exposure** | **Exposure level** | **Cases** | **Person-years** |
| --- | --- | --- | --- |
| Unexposed | — | 15 163 | 2 877 670 |
| Any exposure | — | 25 410 | 4 584 560 |
| Cement dust | Low | 2612 | 481 440 |
|  | High | 537 | 76 457 |
| Concrete dust | Low | 7272 | 1 427 354 |
|  | High | 7798 | 1 420 433 |
| Quartz dust | Low | 7275 | 1 209 833 |
|  | High | 1786 | 236 645 |
| Welding fumes | Low | 168 | 28 799 |
|  | High | 4035 | 715 111 |
| Diesel exhaust | Low | 4152 | 593 671 |
|  | High | 2437 | 345 476 |
| Asphalt fumes | Low | — | — |
|  | High | 922 | 147 273 |
| Wood dust | Low | 2210 | 498 471 |
|  | High | 100 | 17 861 |
| Asbestos | Low | 1386 | 221 366 |
|  | High | 586 | 125 300 |
| MMMF | Low | 2058 | 371 701 |
|  | High | 1129 | 232 444 |
